# Intravenous administration of the conditionally replicative adenovirus Ad5-Δ24RGD induces regression of osteosarcoma lung metastases

**DOI:** 10.1186/1476-4598-7-9

**Published:** 2008-01-23

**Authors:** Harm CA Graat, Victor W van Beusechem, Frederik HE Schagen, M Adhiambo Witlox, Eugenie S Kleinerman, Marco N Helder, Winald R Gerritsen, Gertjan JL Kaspers, Paul IJM Wuisman

**Affiliations:** 1 Department of Orthopedic Surgery, VU University medical center, De Boelelaan 1117, 1081 HV, Amsterdam, the Netherlands; 2 Department of Medical Oncology, VU University medical center, De Boelelaan 1117, 1081 HV, Amsterdam, the Netherlands; 3 Division of Pediatrics, University of Texas, M. D. Anderson Cancer Center, 1515 Holcombe Blvd, Houston, TX 77030, USA; 4 Department of Cancer Biology, University of Texas, M. D. Anderson Cancer Center, 1515 Holcombe Blvd, Houston, TX 77030, USA; 5 Department of Pediatric Oncology/Hematology, VU University medical center, De Boelelaan 1117, 1081 HV, Amsterdam, the Netherlands

## Abstract

Metastatic osteosarcoma (OS) has a very poor prognosis. New treatments are therefore wanted. The conditionally replicative adenovirus Ad5-Δ24RGD has shown promising anti-tumor effects on local cancers, including OS. The purpose of this study was to determine whether intravenous administration of Ad5-Δ24RGD could suppress growth of human OS lung metastases. Mice bearing SaOs-lm7 OS lung metastases were treated with Ad5-Δ24RGD at weeks 1, 2 and 3 or weeks 5, 6 and 7 after tumor cell injection. Virus treatment at weeks 1–3 did not cause a statistically significant effect on lung weight and total body weight. However, the number of macroscopic lung tumor nodules was reduced from a median of >158 in PBS-treated control mice to 58 in Ad5-Δ24RGD-treated mice (p = 0.15). Moreover, mice treated at weeks 5–7 showed a significantly reduced lung weight (decrease of tumor mass, p < 0.05), a significantly increased body weight gain (decrease of disease symptoms, p < 0.005) and a reduced number of macroscopic lung tumor nodules (median 60 versus > 149, p = 0.12) compared to PBS treated control animals. Adenovirus hexon expression was detected in lung tumor nodules at sacrifice three weeks after the last intravenous adenovirus administration, suggesting ongoing viral infection. These findings suggest that systemic administration of Ad5-Δ24RGD might be a promising new treatment strategy for metastatic osteosarcoma.

## Findings

Osteosarcoma is the most prevalent non-hematological primary malignant bone tumor. They can be subdivided by location (medullary and surface) and by grade (high or low grade). The vast majority of high-grade osteosarcoma comprises of conventional osteosarcoma, but also includes high-grade surface, telangiectatic and small-cell osteosarcoma [1]. Patients with high-grade osteosarcoma (OS) without evident metastatic disease at presentation have 5-year survival of about 50–70% achieved by preoperative and postoperative chemotherapy with aggressive surgery [2-6]. Patients presenting with overt metastatic disease undergo the same pre- and postoperative chemotherapeutic regimen with resection of all metastases whenever feasible. The majority of osteosarcoma metastases are located in the lungs [4,7,8]. Multiple lung metastases and bilateral distribution appear to be poor prognostic factors for survival with 5-year overall survival rates of 26% and 20%, respectively; in contrast to 75% for a solitary lung lesion [8]. Moreover, OS patients that relapse after first achieving complete surgical remission during combined-modality chemotherapy have a 5-year overall survival of 23% [9]. Long-term survivors were only observed after a second surgical remission, indicating that metastastectomy of all lesions is essential. The role of second-line chemotherapeutic agents is unsure and might result in limited improved outcome [9]. Clearly, for inoperable OS patients new treatment modalities are needed.

We explore the use of conditionally replicative adenoviruses (CRAds) as a potential new treatment modality for high grade OS and its lung metastases. Virus replication will lyse tumor cells and released viral progeny can infect neighboring tumor cells leading to lateral spread and increased tumor cell kill. Previously we found that primary OS cells express low levels of the high affinity receptor for adenoviruses, the coxsackie and adenovirus receptor (CAR) [10,11]. In contrast, primary OS cells express high levels of integrins [12]. To circumvent CAR deficiency and thereby reduced tumor cell infectivity, we use the CRAd variant Ad5-Δ24RGD that carries a cyclic Arg-Gly-Asp (RGD-4C) integrin binding motif in its fiber knob domain [13]. This CRAd expresses mutant E1A that cannot bind to pRb. Consequently, E2F, essential for viral replication, is not released. Therefore, this CRAd is limited to replicate in cancer cells with constitutively active E2F and not in non-cycling normal cells with functional pRb [14]. In vivo experiments showed that this virus induced tumor regression of subcutaneous glioma, osteosarcoma and cervical cancer tumors after intra-tumoral or systemic administration [12,15-17]. Infection of CAR-deficient OS cells with this integrin-targeted virus resulted in increased infectivity and cell kill. Furthermore, we showed that intra-tumoral administration of Ad5-Δ24RGD in subcutaneous primary OS tumors resulted in a significant tumor growth delay [12].

Local treatment of OS with CRAds has shown promising results in animal models [12,18]. However, the major challenge to cure OS patients depends on an effective treatment for (non-resectable) metastases. Biodistribution studies in animals indicated that a considerable amount of intravenously administered Ad5-Δ24RGD is delivered to the lungs (25% of the liver dose) [17]. In addition, the RGD-modification might partially avoid reduced infectivity as result of neutralizing antibodies [19,20]. In the present study, we explore the use of intravenous administered Ad5-Δ24RGD for the treatment of OS lung metastases.

**Figure 1:**
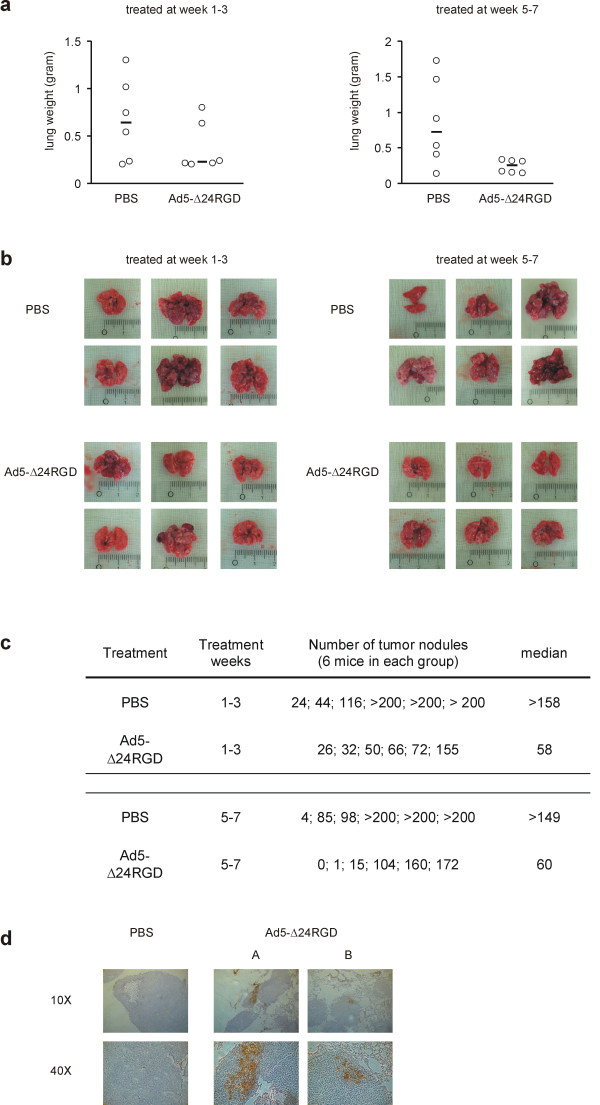
Therapeutic effect of Ad5-Δ24RGD infusion 1–3 (left panel figure a & b) or 5–7 (right panel figure a & b) weeks after SaOs-lm7 injection. (a) Effect of intravenous administration of Ad5-Δ24RGD on OS pulmonary tumor burden. Lungs were removed and weighed. Open circles depict lung weight of each mouse and black lines show the median of each group. (b) Macroscopic appearance of dissected lungs. (c) Number of lung tumor nodules per mouse. (d) Immunohistochemical staining for expression of the adenovirus hexon protein in lungs of mice treated at weeks 5–7. Ten weeks after tumor cell injection and three weeks after the last virus injection lungs were isolated and analyzed for hexon expression. Representative pictures of the lungs from a PBS control mouse and two Ad5-Δ24RGD treated mice (A & B) are shown at 10× and 40× magnification.

We first performed a pilot study in which mice were given 1 × 10^9 ^plaque forming units (pfu) intravenously once a week for three consecutive weeks (3 × 10^9 ^pfu total). This was the practically highest achievable dose and injection of this amount of virus did not affect body weight, suggesting that it did not induce overt toxicity. Therefore, this dose was chosen to explore the anticancer potency against OS lung metastases. SaOs-lm7 cells were injected intravenously into mice to establish OS lung metastases [21]. In a first experiment, mice bearing OS lung metastases were treated with 1 × 10^9 ^pfu Ad5-Δ24RGD or with PBS at weeks 1, 2 and 3 after SaOs-lm7 injection. As parameter of mouse well-being, their relative gain in body weight was calculated. Ten weeks after tumor cell injection, mice were sacrificed and their lungs dissected. Mouse lung weight and number of macroscopic lung tumor nodules were quantified to assess Ad5-Δ24RGD anticancer effect. Mice treated with Ad5-Δ24RGD or PBS showed no significant difference in body weight gain during the experiment (data not shown). As shown in figure 1a, average lung weight of virus treated animals appeared to be decreased in comparison to PBS treated control animals, but this difference was statistically not significant (p = 0.15). Macroscopic appearance of the lungs reflected the lung weight measurements, where a lower lung weight paralleled fewer and smaller lung metastases and a higher lung weight correlated with more and larger lung metastases (figures 1b). In the PBS treated group, lungs often contained numerous lung metastases; sometimes more than could be counted (> 200). The median number of macroscopic tumor nodules on the lungs of PBS treated animals was >158 (range 24 – >200). Lungs of Ad5-Δ24RGD treated animals carried a median number of 58 lung metastases (range 26–155; figure 1c).

In a next experiment, we delayed the onset of oncolytic adenovirus treatment by 4 weeks. Virus or PBS was injected once per week at 5, 6 and 7 weeks after SaOs-lm7 tumor cell injection. Mice treated with Ad5-Δ24RGD showed an average increase in weight of 18%, which was significantly higher than the average 8% weight gain observed for PBS treated control animals (P < 0.005). Moreover, intravenous administration of Ad5-Δ24RGD resulted in a significant reduction of lung weight (median 241 mg) compared to PBS treated control animals (median 723 mg; figure 1a; P < 0.05). The lung weight of virus treated mice was just above normal mouse lung weight (i.e., approximately 180 mg). The higher tumor mass in the PBS treated group suggested that the total body weight gain, after correction for increased tumor mass, differed even more between the PBS and virus treated animals. Also in this experiment, macroscopic appearance reflected lung weight measurements (figure 1b). The median number of macroscopic lung metastases (figure 1c) in PBS treated animals was >149 (range 4 – >200) and in Ad5-Δ24RGD treated animals 60 (range 0 – 172).

To strengthen the hypothesis that intravenous delivered Ad5-Δ24RGD caused an antitumor effect against OS lung metastases, the lungs of mice from the second experiment treated with Ad5-Δ24RGD at weeks 5–7 and dissected at week 10 were analyzed for adenoviral infection by hexon staining. As shown in figure 1d, foci of infected cells were present in SaOs-lm7 lung nodules, but not in normal lung tissue. Thus, adenovirus infection could still be detected in OS lung metastases 3 weeks after the last intravenous Ad5-Δ24RGD injection, supporting the assumption that ongoing virus infection contributed to the beneficial effects of systemic CRAd treatment. Adenovirus-positive foci were not surrounded by apparent fibrosis, suggesting that connective tissue barriers did not hamper intra-tumor spread of the virus. However, such efficacy-limiting physical barriers might exist in OS.

In general, Ad5-Δ24RGD treatment seemed to reduce lung weight and number of macroscopic tumor nodules in both tested treatment schedules. These parameters are commonly used for therapeutic efficacy evaluation. Treatment effects were most evident when virus treatment was applied 5–7 weeks after tumor cell injection. Treatment at week 5–7 also resulted in a significant body weight increase, suggesting improved mouse well-being. More insight into the kinetics of OS lung metastasis growth and response to treatment could be obtained if longitudinal in vivo imaging of SaOs-lm7 tumor growth would be possible. Unfortunately, a derivative reporter cell line for bioluminescent imaging is not available.

Together, our observations suggest that Ad5-Δ24RGD is active against OS lung metastases, which is in line with recent finding by Li *et al *[22], who showed that a transcriptional replication-competent adenovirus (AdOC-E1a) reduced the number of OS lung tumor nodules. Furthermore, our experiments suggest that timing of virus injection influences treatment outcome. A possible explanation for the more effective outcome in mice treated at weeks 5–7 might be found in a dependence on an established tumor vasculature for systemic CRAd delivery. SaOs-lm7 injected tumor cells will form microscopic lung metastases by 3–5 weeks and visible lung nodules by 6 weeks [21]. Microscopic tumors are usually in a pre-vascular state and only growth beyond 2–3 mm^3 ^requires blood vessel formation [23]. Virus injected at week 1–3 thus presumably treats isolated tumor cells or microscopic tumor nodules, which could be reached via diffusion, whereas treatment at week 5–7 would allow vascular delivery to macroscopic tumor nodules. Although we documented significant therapeutic efficacy after three injections of Ad5-Δ24RGD, mice were not completely cured from their OS lung metastases. To increase treatment efficacy extended administration schemes could be tested. In addition, in a clinical setting in humans isolated lung perfusion to increase CRAd delivery could be considered. Several clinical trials delivering therapeutic agents to sarcoma lung metastases via isolated lung perfusion have been conducted [24]. Clinical trials with Ad5-Δ24RGD for the treatment of glioma and ovarian cancer are developed and will be started soon [25]. These studies will provide valuable data on the safety profile of this CRAd in humans important to pursue development of Ad5-Δ24RGD for OS and OS lung metastases treatment.

## Methods

### Cell lines

SaOs-lm7 cells are derived from SaOs-2 human OS cells by repetitive cycling through the lungs of nude mice [21]. SaOs-lm7 cells are grown in Eagle's minimal essential medium supplemented with nonessential amino acids, sodium pyruvate, L-glutamine, 10% fetal calf serum (FCS) and 50 IU/ml penicillin plus 50 μg/ml streptomycin (PS; Life Technologies, Breda, the Netherlands). Cultures are maintained and harvested at sub-confluence. A549 and 293 cells were obtained from the American Type Culture Collection (Manassas, VA) and are maintained in F12-supplemented Dulbecco's modified Eagle's medium (DMEM) with 10% FCS and PS. All cells are cultured at 37°C in a humidified, 5% carbon dioxide atmosphere.

### Recombinant adenoviruses

The CRAd Ad5-Δ24RGD lacks 24 base pairs encoding 8 amino acids in the pRb binding domain of E1A and carries a cyclic RGD epitope in the HI-loop of the fiber [13]. This CRAd was propagated on A549 cells. Virus was purified using cesium chloride gradient banding. Functional titers in pfu were determined by end-point limiting dilution on 293 cells.

### OS lung metastasis model in nude mice

Female athymic/nude/nude mice were purchased from Harlan (Horst, the Netherlands). Animals were maintained under pathogen-free conditions and fed a standard laboratory diet *ad libitum*. All experiments were approved by the local committee on animal experiments and were carried out under the conditions established by the European Community (directive 86/609/CCE). Mice were injected with 5 × 10^6 ^SaOs-lm7 cells in the lateral tail vein in 200 μl Hanks Balanced Salt Solution w/o calcium and magnesium. Intravenous injection of SaOs-lm7 cells results in microscopic lung metastases by week 3–5 and macroscopic disease at 6 weeks.

### Intravenous administration of Ad5-Δ24RGD to mice bearing osteosarcoma lung metastases

In two independent experiments, 12 mice bearing SaOs-lm7 lung metastases were randomly divided into 2 groups of each 6 mice. Mice were treated three times, in weeks 1, 2, and 3 (experiment 1) or in weeks 5, 6 and 7 (experiment 2). The first group received intravenous injections in the tail vein of 1 × 10^9 ^pfu Ad5-Δ24RGD in 200 μl PBS and the second control group received 200 μl PBS.

Body weight was measured twice a week. All mice were sacrificed in week 10 after tumor cell injection. The lungs were removed in toto without difficulty and photographed. Two researchers counted tumor nodules on the outside of the lungs independently and the average of the two measurements was calculated. Lungs were snap frozen in liquid nitrogen and weighed using a Mettler H31 (± 0.1 mg; Mettler-Toledo International Inc).

### Immunohistochemical analysis

Cryosections of lungs obtained at week 10 from mice treated at weeks 5, 6 and 7 after tumor cell injection were fixed in acetone. Immunohistochemical staining for adenovirus (hexon) was done using goat anti-adenovirus antibody 1056 (Chemicon International, Temecula, CA). Sections were then incubated with biotinylated goat anti-rabbit IgG antibodies. The immune reaction was visualized using the streptavidin-biotin-Horse Radish peroxidase complex method (StrepABComplex/HRP; Dako, Glostrup, Denmark) followed by diaminobenzidine solution (Vector Laboratories, Inc., Burlingame, CA). Sections were counterstained with hematoxylin (Sigma Aldrich, St. Louis, MO), dehydrated and mounted.

### Statistical analysis

Differences in relative gain in mouse weight, number of lung metastases and total lung weight between treatment groups were analyzed with a two and one-tailed Mann-Whitney test, respectively, using GraphPad Instat 3.0 (GraphPad Software, Inc., San Diego, CA).

## Competing interests

The author(s) declare they have no competing interests.

## Authors' contributions

HCAG: designed and carried out the experiments and drafted the manuscript

VWB: designed experiments, analyzed data and critically commented on final manuscript

FHES: counted OS lung tumor metastases and corrected manuscript

MAW: optimized mouse model and corrected manuscript

ESK: optimization of mouse model, and commented on manuscript

MNH: designed experiments and corrected manuscript

WRG: supervised project and commented on manuscript

GJLK: obtained funding, supervised project and corrected final manuscript

PIJMW: obtained funding, supervised project and corrected final manucript

All authors read and approved the final manuscript.
